# Potential of Nuclear Imaging Techniques to Study the Oral Delivery of Peptides

**DOI:** 10.3390/pharmaceutics14122809

**Published:** 2022-12-15

**Authors:** Tanya Saxena, Claire Sie, Kristine Lin, Daisy Ye, Katayoun Saatchi, Urs O. Häfeli

**Affiliations:** 1 Faculty of Pharmaceutical Sciences, University of British Columbia, Vancouver, BC V6T1Z3, Canada; 2 Department of Pharmacy, Faculty of Health and Medical Sciences, University of Copenhagen, 2100 Copenhagen, Denmark

**Keywords:** oral delivery, peptide, gastrointestinal imaging, SPECT, pre-clinical imaging

## Abstract

Peptides are small biomolecules known to stimulate or inhibit important functions in the human body. The clinical use of peptides by oral delivery, however, is very limited due to their sensitive structure and physiological barriers present in the gastrointestinal tract. These barriers can be overcome with chemical and mechanical approaches protease inhibitors, permeation enhancers, and polymeric encapsulation. Studying the success of these approaches pre-clinically with imaging techniques such as fluorescence imaging (IVIS) and optical microscopy is difficult due to the lack of in-depth penetration. In comparison, nuclear imaging provides a better platform to observe the gastrointestinal transit and quantitative distribution of radiolabeled peptides. This review provides a brief background on the oral delivery of peptides and states examples from the literature on how nuclear imaging can help to observe and analyze the gastrointestinal transit of oral peptides. The review connects the fields of peptide delivery and nuclear medicine in an interdisciplinary way to potentially overcome the challenges faced during the study of oral peptide formulations.

## 1. Introduction

One of the most common routes of drug administration is oral delivery, 53% of FDA-approved drugs from 2015–2020 [1] since it provides better patient compliance and low manufacturing cost [2,3]. While parenteral drug delivery typically shows 80–100% bioavailability, rapid onset of drug effect, and most predictable pharmacokinetics, about 5% of the world’s population is still needle-phobic, and injections generally require medical training for administration [4]. Thus, oral drug delivery is by far the preferred route of administration by patients [5]. It plays a major role in the treatment of chronic disorders such as diabetes, gut infections, irritable bowel syndrome, and hypertension that require daily administration [6]. Despite many benefits, oral peptides comprise only 4% of protein and peptide FDA-approved formulations, primarily due to the challenges faced during the development of oral delivery systems [7]. The harsh environment of the gastrointestinal tract comprising acidic pH, enzymes, mucus lining, and gut microbiome prevents the delivery of sensitive drugs, e.g., peptides, into the systemic circulation, thereby reducing their oral bioavailability [8,9].

Peptides are polymeric biomolecules consisting of approximately 2–50 amino acids linked by peptide bonds [10]. One of the most commonly administered therapeutic peptides is insulin, a 51-amino acid long peptide hormone secreted by beta cells in the pancreas [11]. Insulin is used to treat diabetes which affected 537 million adults worldwide and caused 6.7 million deaths in 2021 [12]. In the past, insulin injections were given subcutaneously to treat diabetes due to low oral bioavailability in the gastrointestinal (GI) tract [13]. However, in 2019, a clinical Glucagon-like-peptide-1 (GLP-1) receptor agonist called Rybelsus^®^ (Semaglutide) came into the market that treats Type 2 diabetes by stimulating the secretion of insulin from the pancreas [14]. It shows better efficacy compared to other GLP-1 agonists but has very low bioavailability (~1%) and can cause pancreatitis or thyroid cancer [15,16].

Peptides are very versatile biopharmaceuticals as they exhibit high specificity, bind to a variety of drug targets, and express low toxicity [17]. Nevertheless, their limitations exceed their benefits as they show low physiochemical stability, undergo proteolysis, and may induce immunogenicity [18]. Furthermore, high molecular weight, low lipophilicity, and the presence of charged functional groups make them poor permeators and reduce their bioavailability further [7,8,19,20,21,22].

**Figure 1:**
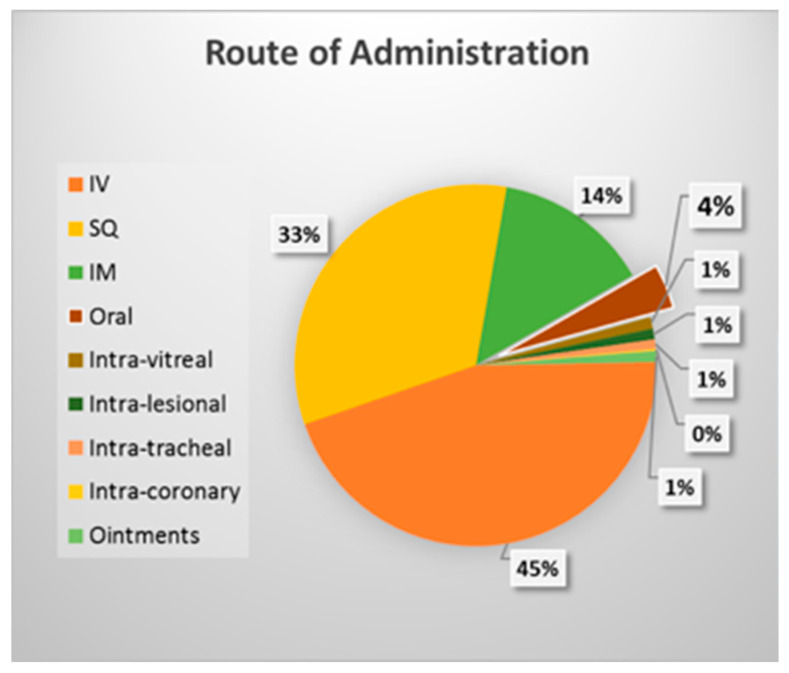
Pie charts display the current distribution of FDA-approved peptide drugs based on their route of administration [7].

The low oral bioavailability of peptides caused by these barriers has adversely affected the approval of oral peptide formulations, as seen in Figure 1 [7]. In July 2017, there were 380 drug variants (chemically modified analogs) of the total Federal Drug Administration (FDA) approved peptides in clinical use. Only 13 of these drug variants were delivered orally, while almost half (158) of them were administered intravenously, 116 were given subcutaneously, and 49 were intramuscularly [7]. Orally delivered peptides thus comprise only 4% of total peptide use (Figure 1) [7]. However, there has been a recent increase in the approval of oral peptide formulations due to innovative interdisciplinary drug delivery research. Currently, in 2021, there are 26 FDA-approved oral formulations of peptides, including the already mentioned 13 peptides plus octreotide and their drug variants (Table 1) [23]. Similarly, the European Medical Agency (EMA) approved 18 peptide drugs between 2010 and 2019, of which only four were administered orally, and two were retracted recently [23]. It is thus very apparent that oral delivery of peptides is still an uncommon method of clinical administration.

### 1.1. Barriers to Oral Delivery of Peptides

**Figure 2:**
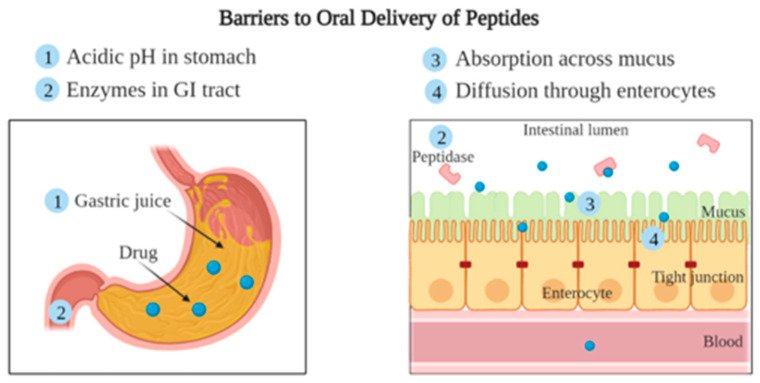
Barriers to oral delivery of peptides.

The first barrier for peptides (Figure 2) is potential degradation by gastric juices in the stomach, which is low in pH (~1 to 3) and high in proteases, e.g., pepsin [40]. As peptides leave the stomach, the pH gradually increases in the small intestine (5.5–7), large intestine (5.5–7.5), and colon (6.5–7.5) [3,41]. These changes in pH can chemically and functionally alter the 3D structure of peptides. The next barrier is enzymes such as trypsin and chymotrypsin present in the intestine, especially in the first segment of the small intestine (duodenum), that degrade peptides into small non-functional fragments. The thick mucus covering the intestinal wall acts as the third barrier and hinders the drug uptake process. Underlying the mucus is the last barrier made up of special epithelial cells called enterocytes that allow selective permeation of molecules into the systemic circulation but overall reduce drug penetration through the intestinal membrane [8].

Protease inhibitors, permeation enhancers, and polymeric encapsulation are some of the approaches that improve the pharmacological effect and oral bioavailability of peptides [25,42]. For example, Tarsa’s oral recombinant salmon calcitonin (TBRIATM) has shown encouraging safety and efficacy results in Phase 3 ORACAL trial for the treatment of postmenopausal osteoporosis by incorporating the approaches mentioned above. The tablet contains an outer layer of pH-lowering agent (citric acid) and an inner layer of peptide calcitonin along with the permeation enhancer lauroyl l-carnitine. An enteric coating of hydroxypropylmethylcellulose helps bypass the acidic stomach pH to achieve intestinal delivery. TBRIATM was less immunogenic but similarly effective and safe compared to commercially available nasal calcitonin sprays [43].

Nanoparticle and microsphere encapsulation is also effective in protecting peptides during oral delivery and increasing pharmacological effect [8]. For example, researchers have shown that the peptide insulin loaded in microspheres using a 1:1 ratio of poly(fumaric anhydride) and poly(lactide-co-glycolide) successfully penetrated the intestinal epithelium in rats and promoted the glucose uptake in cells, while the controls (saline or insulin solution given orally) increased blood glucose as expected to non-significantly different levels from each other [44]. 

### 1.2. Observing Oral Delivery of Peptides

Although oral delivery has been well studied clinically, scientists still struggle to accurately quantify drug uptake and distribution in the GI tract pre-clinically due to the smaller size and dimensions of the organs in animals [45,46]. In addition, physiological and anatomical barriers such as gastric juices, enzymes, and intestinal mucus membranes present in the GI tract reduce the bioavailability of drugs considerably [2,5]. Peptides, such as other biomolecular drugs, have sensitive 3D structures that get degraded easily in the GI tract and thus are not delivered orally (further discussed in the next section) [5,47,48]. Drug delivery systems (DDS) have been formulated to overcome this degradation by protecting/encapsulating peptides in excipients that improve the amount of intact functional peptides reaching the systemic circulation after oral administration [17,47,49,50]. 

The amount and rate of drug delivered from the oral DDS to the systemic circulation, i.e., the pharmacokinetic properties of the DDS, can be studied using different techniques such as traditional biodistribution studies, histological analyses, blood sampling techniques, and more recently, optical and fluorescent microscopy, and nuclear imaging techniques [51,52,53,54]. 

Over a period, new techniques have emerged, overcoming the limitations of earlier techniques, but each technique has its advantages and limitations. Biodistribution studies give an accurate quantitative analysis of drug distribution in the body but require a large number of animals for each time point to study drug uptake in different organs over time [55]. Whereas histology provides drug uptake at specific locations such as tissue layers at different time points after sacrificing the animals, thereby requiring discrete animals for each data point leading to large standard deviations [56,57,58]. Blood sampling overcomes this challenge since blood can be collected from the same animal at different time points, but it does not provide any data regarding drug uptake in tissues or organs. On the other hand, microscopy provides a good understanding of cellular uptake and reduces animal variability but lacks the depth of penetration [59]. Nuclear imaging overcomes all these challenges through real-time 3D images. It clearly shows the depth of penetration of drug uptake in the same animal since the drug is conjugated with a radioisotope that can be detected on a gamma camera while the drug is moving through the body [59,60,61]. However, nuclear imaging has its own drawbacks, such as low resolution at the cellular level (limitation of current gamma cameras) and the difficulty in observing the stability of radioisotope-bound peptides during GI transit and uptake into circulation [62].

This review, in addition to giving an update on the current clinically available oral peptide formulations and drug delivery systems, also discusses the potential of nuclear imaging techniques to study the oral biodistribution of biopharmaceutical peptide drugs with a special focus on Single Photon Emission Computed Tomography (SPECT) and Positron Emission Tomography (PET) imaging, as they are the most sensitive and quantitative in vivo nuclear imaging methods. 

## 2. Existing Systems of Oral Delivery of Peptides

**Figure 3:**
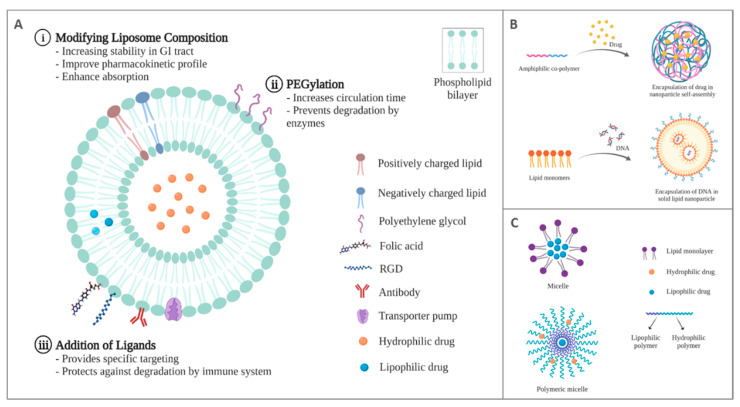
Schematic diagram of currently used drug delivery systems for oral delivery of peptides. (**A**). Liposome with possible physicochemical modifications: (i) Lipid composition of liposomes can be modified to include varying ratios of positive, negative, and neutral phospholipids, (ii) PEGylated phospholipids increase steric stability, (iii) Ligands such as antibodies, carbohydrates, and proteins can be added to the surface of liposomes for stability or specific targeting of a delivery system; (**B**). Self-assembled and solid lipid nanoparticles; (**C**). Micelle and polymeric micelle.

As mentioned briefly in the section above, to overcome physiological barriers present in the GI tract, components such as enzyme inhibitors, enteric coatings, and permeation enhancers are employed in DDSs such as liposomes, nanoparticles, micelles, and hydrogels (Figure 3) [47]. While microspheres and dendrimers are also being investigated pre-clinically as possible carriers for oral delivery of peptides [63,64,65,66,67], they are not discussed in this review.

### 2.1. Liposomes

First elucidated by Alec D Bangham in 1961, liposomes are the most common and well-studied type of nanocarrier used for drug delivery [68]. They are defined as vesicles composed of one or more phospholipid bilayers that self-assemble to enclose an aqueous interior space, typically between 50–150 nm in diameter (Figure 3A) [63]. Researchers’ fascination with the oral administration of therapeutic molecules via liposomes began in the 1970s incorporating insulin for managing blood glucose levels [57,69]. Patel and Ryman (1976) showed that liposomal formulations made up of lecithin, cholesterol, and diacetyl phosphate could successfully deliver insulin orally, reducing the blood glucose level of diabetic rats to approximately one-third of its initial value 3 h after administration [11]. They concluded that adopting a liposomal drug delivery system allowed insulin. A peptide previously believed to lose its therapeutic effects when orally administered to survive the harsh environments of the GI tract. Although the results were promising, it was later found that they were neither reproducible nor consistent, with only 54% of spontaneously diabetic rats and 67% of alloxan-induced diabetic rabbits responding to treatment [70]. These inconsistencies may have been in part due to the challenges faced by the early liposomal delivery systems that resulted in low circulation time due to uptake by the reticuloendothelial system and opsonization [71].

There are several advantages of using liposomes for the oral delivery of peptides. First, liposomes are biocompatible due to their lipid composition being similar to biological membranes [72]. Additionally, they are unparalleled among nanocarriers in their ability to encapsulate both hydrophobic and hydrophilic drugs, allowing for a diverse range of therapeutic molecules to be shielded from the external environment and successfully delivered [73]. Furthermore, they can be improved with a wide variety of physicochemical and biophysical functionalities, such as targeting ligand molecules, PEGylation, and polymeric coatings that might enhance peptide delivery and reduce the barriers faced by other oral DDS (Figure 3A) [74,75]. A pioneering liposomal polymerization technique introduced by Dr. Robert Langer’s lab composed of cross-linked 1,2-di (2,4-octadecadienoyl) phosphatidylcholine showed 50% more gastrointestinal stability than regular cholesterol-based liposomes and could deliver 75% of its contents intact to the intestine [76].

Overall, liposomal delivery systems are able to stabilize and protect therapeutic compounds from enzymatic degradation, prevent premature inactivation, and increase absorption through the GI tract [74]. 

### 2.2. Nanoparticles

Nanoparticles are particles typically sized from 1 to 100 nm and made from inorganic materials, such as silicates, carbon, silver, iron, titanium, and cerium, or organic materials, such as lipids, polymers, and proteins [77]. Nanoparticles have multiple benefits, such as efficient delivery of cargo to the blood circulation and the lymphatic system, thereby increasing the drug’s circulation time and concentration at the target site, for example, a. tumor (Figure 3B) [8]. Nanoparticles protect peptides during oral delivery by encapsulating biomolecules inside their core and preventing mechanical or physiological damage to the peptide [78]. Furthermore, bioadhesive nanoparticles assist in increasing the retention and residence time of peptides at target sites, such as under the tongue or in the gastrointestinal tract, increasing their uptake. In particular, mucoadhesive polymers such as chitosan, Carbopol, polymethacrylates, and carboxymethyl cellulose, mixed with or coated onto nanoparticles, promote strong adhesive interactions with mucus and increase the interaction with the intestinal epithelium [8,79]. This prolongs nanoparticles’ time in the intestinal lumen, increases a drug’s concentration in the systemic circulation, and reduces plasma fluctuations and side effects, thus overall improving drug bioavailability and efficacy [63]. 

As an example of nanoparticles used for oral peptide delivery, Zhang et al. in 2006 showed that insulin could be successfully delivered orally via solid lipid nanoparticles with a bioavailability of 4.99% to 7.11% in rats [80]. Another report by Sung et al. confirmed these findings and showed that nanoparticles sized between 110 nm to 250 nm and composed of chitosan and poly (γ-glutamic acid) incorporating 15% insulin resulted in an oral bioavailability of 15.1 ± 0.9% and a visible decrease in blood glucose levels of diabetic rats [81,82]. Mucoadhesive polymers such as chitosan contribute to the opening of tight junctions between the epithelial cells of the lining of the small intestine [82]. However, practical applications of chitosan are limited as it is poorly soluble in the intestine, and chemical modifications to its structure can affect the functionality of peptides being delivered [83]. Another type of polymeric nanoparticles composed of poly(-ε-caprolactone) and Eudragit^®^ RS was found to be more biocompatible and effective but showed low oral bioavailability of 13% in diabetic rats after oral delivery [84]. 

### 2.3. Micelles

Polymeric micelles are spherical structures composed of amphiphilic copolymers which self-assemble in aqueous solutions (Figure 3C) [85]. They are typically under 100 nm in size and possess a hydrophobic core into which lipophilic therapeutic compounds may be loaded [85]. They protect incorporated drugs from degrading factors during circulation, and their altered physicochemical properties can increase membrane permeability [86]. In addition, micellar DDS avoid clearance by the reticuloendothelial system due to their small size and improve its systemic exposure [87]. 

Most successfully, micellar delivery systems were experimentally tested and revealed to be applicable for both ocular drug delivery [88] and delivery of anti-cancer drugs [89]. A wide variety of formulated copolymers have also been tested for use in oral drug delivery. For example, polyanionic copolymer mPEG-grafted-alginic acid (mPEG-g-AA)-based polyion complex (PIC) micelles were shown to increase the intestinal permeability of the 32-amino-acid peptide salmon calcitonin by 2.24-fold across a Caco-2 monolayer without affecting the cell integrity [90].

In 2020, for the first time, Han et al. developed zwitterionic micelles that can successfully deliver oral insulin in animals [91]. The micelles made up of DSPE-PCB (DSPE-1, 2-distearoyl-sn-glycero-3-phosphoethanolamine, PCB-poly(carboxybetaine) mimic the surface of capsid viruses, thereby penetrating the epithelial cell layer of GI tract by proton-assisted amino acid transporter 1 (PAT1) [92,93,94]. A key benefit of these micelles is their ultra-low critical micelle concentration (CMC) of ~10^-6^ mM that prevents the tight junctions from opening [95]. Thus, peptides delivered in DSPE-PCB micelles are only delivered by transcellular pathway and show a unique safety profile by maintaining gut health. The researchers also fabricated a prototype of oral insulin by encapsulating freeze-dried DSPE-PCB/insulin in an enteric-coated capsule and administering it in diabetic rats [91]. The formulation showed an oral bioavailability of 42.6% and a prolonged hypoglycemic effect of up to 6 h, depending on the loading ratio of DSPE-PCB and insulin [91].

### 2.4. Hydrogels

Another DDS under consideration for oral drug delivery of peptides is hydrogels. They are defined as polymers consisting of hydrophilic components cross-linked together, forming a 3D structure that can swell and hold a large amount of water [96]. In a comprehensive review by Sharpe et al., the authors noted that hydrogels are good candidates for oral drug delivery due to their biocompatibility, ability to be modified, and the wide range of materials, either synthetic or natural, that can be used in their fabrication [97]. A range of hydrophilic polymers is used to cross-link and form mesh-like structures that can be loaded with different drug moieties. An advancement in this field comes in the form of smart polymeric composite carriers such as carboxylated chitosan grafted nanoparticles (CCGN) combined with bilaminated films consisting of a hydrophilic alginate-Ca^2+^ mucoadhesive layer and a hydrophobic ethylcellulose backing layer [98]. Calcein was entrapped in the nanoparticles and loaded into the hydrogel alginate film. In vitro release studies showed no release in simulated gastric fluid at pH 1.2 (similar to humans) and 100% release within 30 min at intestinal pH due to the complete dissolution of the alginate layer [98]. 

## 3. Nuclear Imaging

To study the movement and ability of DDS to reach the target site, scientists have invented a variety of imaging techniques ranging from optical techniques to nuclear imaging. Optical techniques include light, electron, bioluminescence, and fluorescence microscopy for cells, in vivo imaging systems (IVIS) (includes fluorescence and bioluminescence imaging), and optical coherence tomography (OCT) for tissues/animals [99]. The optical techniques provide an easy, cost-effective in vitro quantification of the drug concentration within cells [100]. However, pre-clinical optical imaging is often challenging due to a lack of light absorption into the tissue/organ, making the imaging non-quantitative. The shortcomings of the optical techniques can be overcome by nuclear imaging, also known as radionuclide scanning, as it provides real-time analysis, high resolution, and fully quantitative results in 3D [59]. This section discusses the current applications and future potential of nuclear imaging in the field of oral peptide delivery for pre-clinical and clinical studies.

Nuclear medicine imaging, by the use of radiotracers and different radioisotopes, identifies abnormalities in the body at molecular levels, such as cancer, infectious and inflammatory disorders, neurodegenerative conditions, amongst others [59,101]. The tracers are administered intravenously or orally in millicurie (1 mCi = 37 MBq) doses. They accumulate in target organs over time, and through the emission of gamma, beta rays, or positrons, they can be detected by gamma camera systems or PET systems to display an image of the diseased site [102]. Nuclear imaging scans include gamma camera systems and PET systems. They provide a non-invasive, real-time imaging modality that can be validated with an ex vivo biodistribution study wherein the organs are harvested, and radioactivity is measured in each organ at different time points throughout the study or at the end of an experiment [103,104]. A recent advancement in the field of nuclear imaging is fusion imaging, consisting of PET/SPECT imaging combined with Computed Tomography (CT) or Magnetic Resonance Imaging (MRI) which allows the concurrent assessment of both functional/physiological and morphological/anatomical conditions in a patient. Thus, a dual-modality imaging system such as PET/CT, PET/MRI, and SPECT/CT provides the radiation signal overlaid on an accurate visual background of a patient’s target organs and anatomy [60].

**Figure 4:**
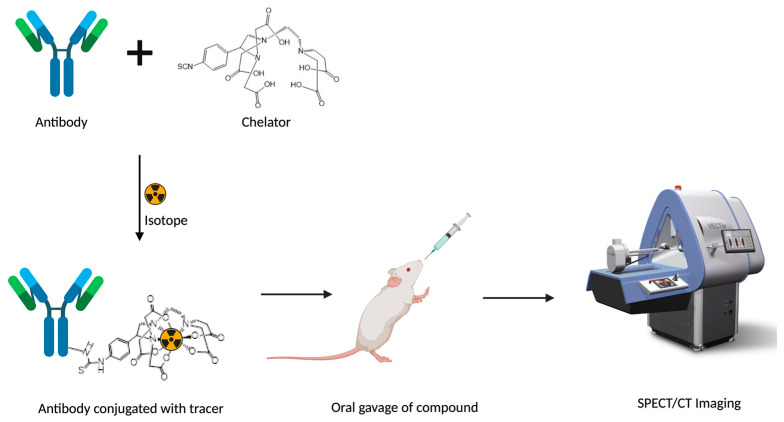
Conjugation of the drug molecule, e.g., an antibody, to a radioisotope using a chelator followed by oral gavage of the radiopharmaceuticals and biodistribution study in mice using SPECT/CT imaging in a preclinical scanner.

The steps toward the clinical development of a radiolabeled peptide for optimal targeting, as seen in Figure 4, include (1) Identification of the receptor or molecular target. Receptor homogeneity, density, and incidence play an important role in targeting receptors. (2) To improve the biological half-life of the peptides, occasionally, a more metabolically stable peptide analog is synthesized, which preserves the functionality and molecular structure of the natural peptide. (3) Radiolabeling process. The peptide is covalently coupled, with or without a spacer and/or chelator that can bind a radioisotope (e.g., ^18^F) or a radiometal (e.g., ^111^In) accordingly. For example, the peptide can be directly conjugated to radioiodine or ^18^F using a prosthetic group with a high labeling efficiency (>95%) and specific activity. (4) In vitro radiopeptide binding to study the affinity of the altered radiolabeled peptide to bind to cell receptors and produce the desired effect. (5) In vivo biodistribution and imaging to study the pharmacokinetic and pharmacodynamic properties of the radiopeptides in established animal models. (6) Testing of successful radiopeptide candidates for safety and efficacy in at least two animal models before going through clinical trials in humans [105,106].

**Figure 5:**
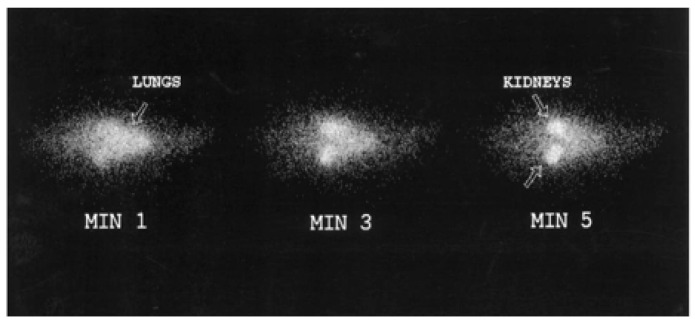
In vivo distribution of ^131^I-GLP-1 in a rat shortly after intravenous administration. Reprinted with permission from Hassan et al. (1999) [41].

One of the first radionuclide imaging studies on the (non-oral) delivery of peptides was carried out by Hassan et al. in 1999 [41] and showed the biodistribution of Iodine-131 (^131^I) radiolabeled Glucagon-like-Peptide-1 (GLP-1) (7–36) in rats after intravenous administration (Figure 5) using a gamma camera. It confirmed a very short half-life (~3.3 min), a high clearance rate (~117 mL/min) of GLP-1 and showed major accumulation in the kidneys after enzymatic degradation. 

Clinically, radiolabeled somatostatin analogs are successfully used for cancer therapy after intravenous injection. For example, Somakit TOC is a diagnostic medicine approved by EMA, used to image gastroenteropancreatic neuroendocrine tumors (GEP-NETs). The kit contains the somatostatin analog, edotreotide, that binds to somatostatin receptors present in the GI tract and pancreas. The peptide is radiolabeled with the PET isotope gallium-68 before injection so that specific cancer cell receptor binding can be imaged. After confirming cancer diagnostically, Lutathera is used to treat GEP-NETs by irradiating the tumors with the beta-emitter lutetium-177. For this purpose, the somatostatin analog DOTA-TATE is radiolabeled with ^177^Lu to form a radiopeptide that specifically binds to the somatostatin receptors present in the tumor cells. While these radiolabeled peptides have been available commercially for a while now, no oral formulation has been reported.

### 3.1. Single Photon Emission Computed Tomography (SPECT) Imaging

Due to multiple barriers faced during the oral delivery of peptides, as stated in Section 1.1, the real-time imaging of peptides reaching the target site requires high sensitivity. SPECT imaging provides this high sensitivity and is additionally a great alternative to optical imaging, as it provides truly quantitative detection of radioactivity in comparison to the easily attenuated fluorescent or bioluminescent probes in live animals. For example, Niu et al. developed a nanocomplex between insulin as the model peptide and octaarginine as a cell-penetrating peptide, enveloped by the protecting polymer poly (glutamic acid)-poly (ethylene glycol) (PGA-PEG) that provided intestinal stability and much penetration [107]. Octaarginine was chemically conjugated with cholesterol or lauric acid to increase electrostatic/hydrophobic interaction with insulin. In vitro studies using a Caco-2 cell monolayer showed high cellular uptake of insulin (47.6 ± 5.8%) into epithelial cells. However, only 2.1% of the radioactive insulin went across the Caco-2 cell line monolayer [107]. 

**Figure 6:**
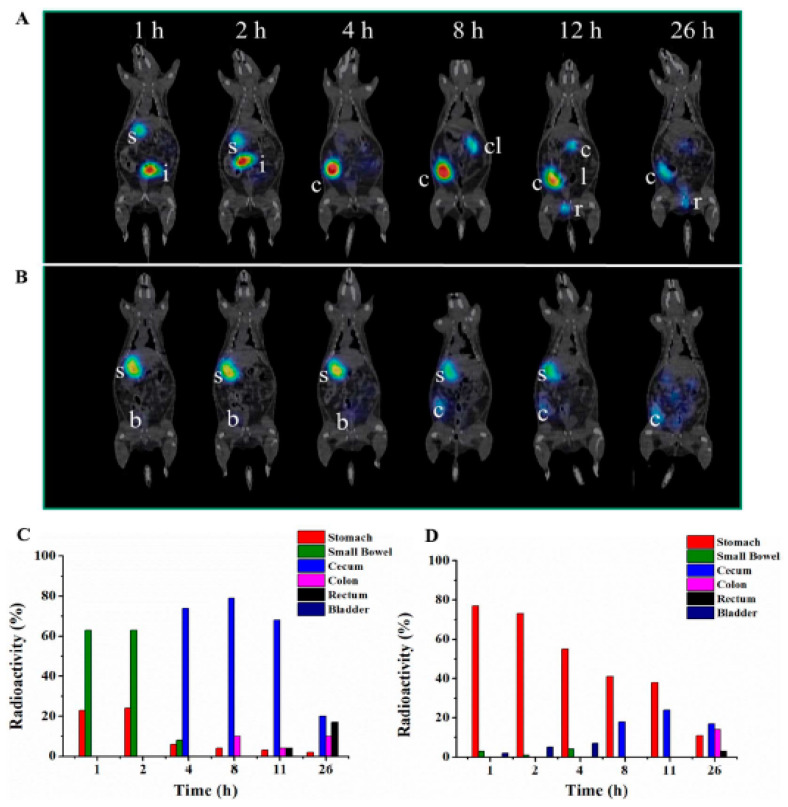
Oral biodistribution of ^99m^Tc-enveloped nanocomplexes (ENCPs) and ^99m^Tc pertechnetate (control) at 1, 2, 4, 8, 11, and 26 h using SPECT/CT imaging (**A**,**B**) and gamma counting (**C**,**D**) in Wistar rats (s = stomach, i = intestine, c = cecum, cl = colon, r = rectum, b = bladder). Reprinted with permission from Niu et al. (2018) [107].

Despite the low penetration, the researchers performed an in vivo pharmacokinetic study in Wistar rats using SPECT imaging (Figure 6). The authors radiolabeled the PGA-PEG-conjugated insulin nonspecifically with 99% labeling efficiency on the polymer end, using stannous chloride using pertechnetate (^99m^TcO_4_^−^). GI transit across the small intestine between 2 to 4 h and accumulation in the cecum for 11 h were observed [107]. Although the imaging method was not sensitive enough to measure the nanocomplex in the systemic circulation, the GI transit of the radiopharmaceutical could be studied properly over time, with the caveat that it likely formed ^99m^Tc-oxides possibly not reflecting the fate of insulin. Furthermore, glucose levels in the blood did not change, pointing to minimal delivery of intact insulin into the systemic circulation.

**Figure 7:**
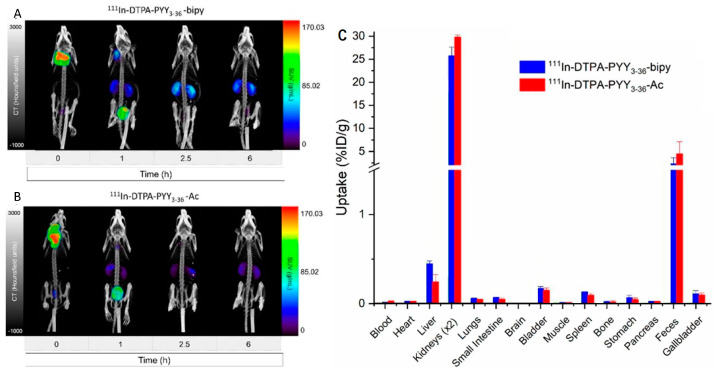
Coronal view of SPECT/CT scans after subcutaneous administration of (**A**) ^111^In-DTPA-PYY3–36-bipy and (**B**) ^111^In-DTPA-PYY3–36-Ac in mice (n = 3). (**C**) Biodistribution (%ID/g) of radioconjugates 24 h post-injection. Reprinted with permission from Kalomoiri et al. (2020) [108].

More recently, the in vivo biodistribution of the peptide PYY3-36 radiolabeled with ^111^In (Table 2) was determined in mice after subcutaneous administration using non-invasive SPECT/CT imaging. The organ-specific uptake of acetylated PYY3-36 activity over 24 h was compared to PYY3-36 functionally modified with a bipyridine (bipy) group. The standardized uptake value (SUV) in the kidneys and injection site after 1 h was found to be double in the case of PYY3-36-bipy (23.4 ± 2.6%) compared to acetylated PYY3-36 (11.3 ± 2.5%) (Figure 7) [108]. 

**Figure 8:**
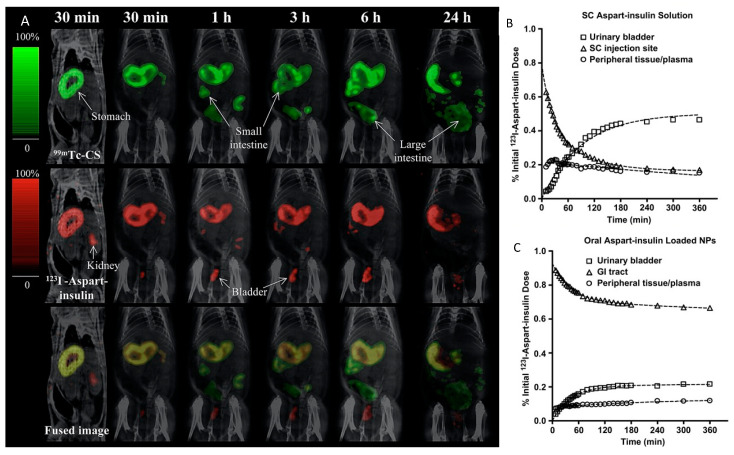
(**A**) Oral biodistribution of ^99m^Tc-labeled-chitosan nanoparticles (green) containing ^123^I-labeled aspart-insulin (red) using SPECT/CT imaging in a rat model. Percentage dose of aspart-insulin observed over time in the urinary bladder, injection site/GI tract, and peripheral tissue/plasma after (**B**) subcutaneous injection and (**C**) oral gavage in rats. Reprinted with permission from Sonaje et al. (2010) [55].

In addition to real-time imaging, nuclear imaging also provides an opportunity for studying the pharmacokinetics of a peptide and its carrier at the same time, whereby one isotope (e.g., ^67^Ga) is conjugated to the peptide and a second isotope with a different energy peak (e.g., ^111^In) is conjugated to the carrier. This allows for a co-localization study of the entire drug delivery system and verifies the in vivo stability of both the peptide and the carrier. An example of this was illustrated by Sonaje et al. in 2010 with dual-isotope imaging, wherein the biodistribution of ^99m^Tc-labeled chitosan as drug carrier and ^123^I-labeled as part-insulin as the drug was studied. The DDS was orally administered in rats as a nanoparticle formulation [55]. Using SPECT/CT imaging, the researchers observed the permeation of the radiolabeled aspart-insulin (analog of natural insulin) from the stomach into the systemic circulation, eventually leading to an accumulation in the kidneys and bladder 30 min after administration. However, the ^99m^Tc-labeled chitosan (carrier) moved through the GI tract and remained in the intestinal lumen. The superimposition of both images shows the ability of SPECT/CT imaging to study the anatomical and functional properties of the DDS (Figure 8).

**Figure 9:**
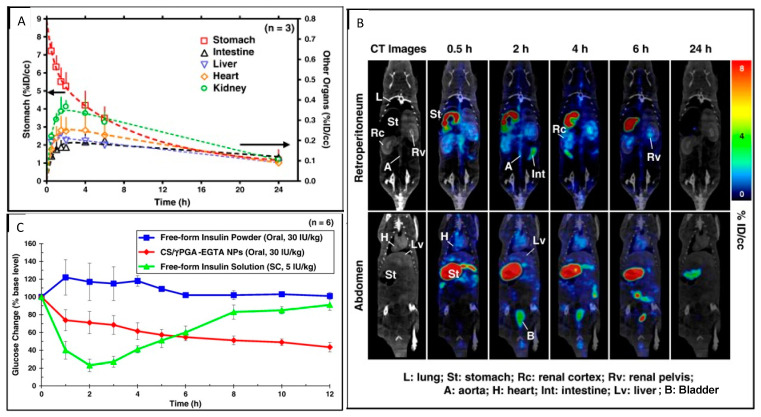
(**A**) Organ activities and (**B**) SPECT images of orally ingested ^123^I-labeled insulin loaded in nanoparticles, superimposed with soft-tissue contrast CT images at different time points; (**C**) Blood glucose change vs. time profiles of diabetic rats treated with different formulations of insulin. Oral gavaged ^123^I-labeled insulin loaded in nanoparticles are in red. Reprinted with permission from Chuang et al. (2013) [110].

The same research group conducted another study in 2013 where they modified the previous nanoparticle formulation by conjugating chitosan to ethylene glycol tetraacetic acid (EGTA) [109] and encapsulated insulin within these nanoparticles to increase its oral bioavailability by 21% [110]. EGTA is known to chelate Ca^2+^ and provide protection to insulin from proteases such as trypsin and chymotrypsin, which are Ca^2+^ dependent enzymes and present inside the intestinal tract [111,112]. A decrease in Ca^2+^ also leads to a reversible opening of the apical junctional complex, thereby increasing the paracellular permeability of insulin through the Caco-2 cell monolayers [113]. The researchers showed that orally delivered ^123^I-labeled chitosan-EGTA nanoparticles circulated through the heart, renal cortex, renal pelvis, and liver leading to a prolonged hypoglycemic effect (~5 h) in diabetic rats (Figure 9) [110]. 

### 3.2. Positron Emission Tomography (PET) Imaging

For pre-clinical research, SPECT provides a slightly higher resolution (~1 mm) and more isotopes for radiotracer chemistries. However, for clinical imaging, PET scanners have a better spatial resolution (3–5 mm) compared to SPECT scanners (5–12 mm) [119]. Moreover, PET isotopes offer better image quality at lower injected radioactivity doses due to higher sensitivity and shorter half-lives of the radionuclides [120]. Thus, PET imaging is a commonly used clinical imaging technique to diagnose tumors, infections, and neurological function [121]. 

**Figure 10:**
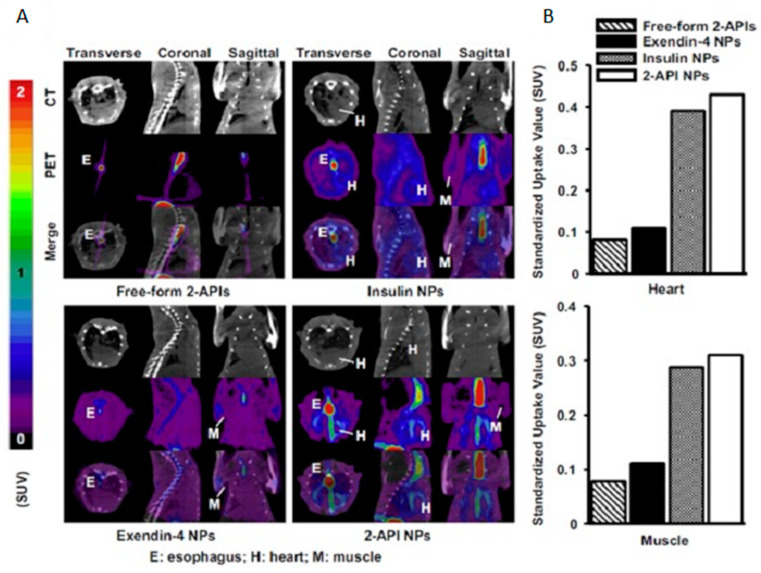
Oral biodistribution and utilization of ^18^F-FDG was studied in type 2 diabetic rats using (**A**) PET/CT imaging and (**B**) standard uptake value (SUV) measurements in the heart and skeletal muscles. 2-API NPs are the combination nanoparticles of insulin and exendin-4. Reprinted with permission from Chuang et al. (2013) [126].

One of the most exploited PET radiopharmaceutical is glucose fluorinated with the PET-isotope Fluorine-18 (^18^F-FDG), which functions as a glucose analog and is used to diagnose cancer [122,123], inflammation and infection [124], and neurodegenerative diseases such as dementia [125]. ^18^F-FDG displays changes in glucose metabolism and tissue accumulation in both animals and humans. Chuang et al. have developed an oral combination therapy with nanoparticles loaded with insulin and exendin-4 (an analog of GLP-1) to treat type 2 diabetes in rats [126]. When released from the nanoparticles, the drugs stimulate the glucose transporter 4 to translocate from its intracellular location to the plasma membrane and facilitate glucose diffusion into the cells. The researchers used ^18^F-FDG to image and quantify the glucose uptake into the heart and muscle 2 h after oral ingestion of the combination nanoparticles. Dynamic PET scans (Figure 10) show high utilization of ^18^F-FDG in the nanoparticles containing insulin or a combination of insulin and exendin-4, validating the pharmacodynamic efficacy of these nanoparticle DDS [126].

**Figure 11:**
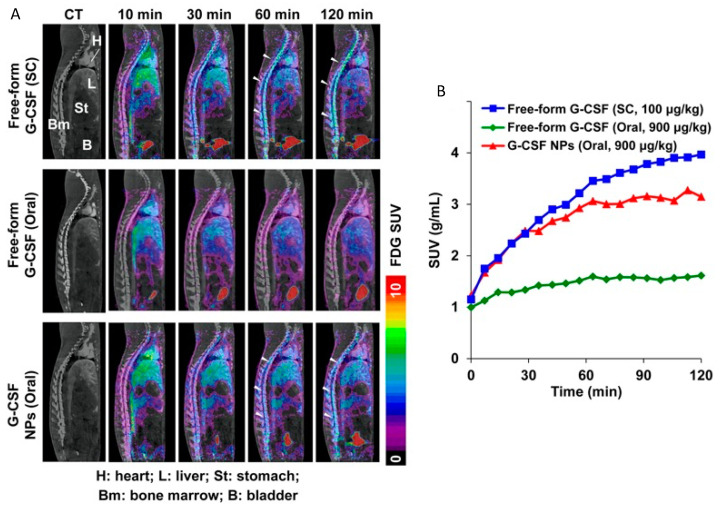
(**A**) PET/CT images show ^18^F-FDG accumulation in rats following G-CSF treatment given subcutaneously (SC), orally, or orally within nanoparticles (NPs). (**B**) Radioactive ^18^F-FDG concentrations are graphed over 2 h in the bone marrow for the different application modalities. Reprinted with permission from Su et al. (2014) [107,128].

^18^F-FDG PET imaging has also been used to study the effects of different small proteins after oral administration [127]. For example, chemotherapy-induced neutropenia can be reduced and recovery accelerated in cancer patients by orally administering granulocyte colony-stimulating factor (G-CSF) in chitosan nanoparticles. As an increased glucose uptake is often observed in the bone marrow during and after G-CSF therapy, Su et al. investigated this in rats after giving the drug subcutaneously, orally, and orally encapsulated in nanoparticles (Figure 11A) [128]. Packing G-CSF into nanoparticles and giving them orally increased the glucose metabolism in the bone marrow more than two-fold over the orally given free G-CSF (Figure 11B) [128]. However, a significantly larger (9-fold) dose had to be given orally to reach similar effects to the subcutaneous administration.

**Figure 12:**
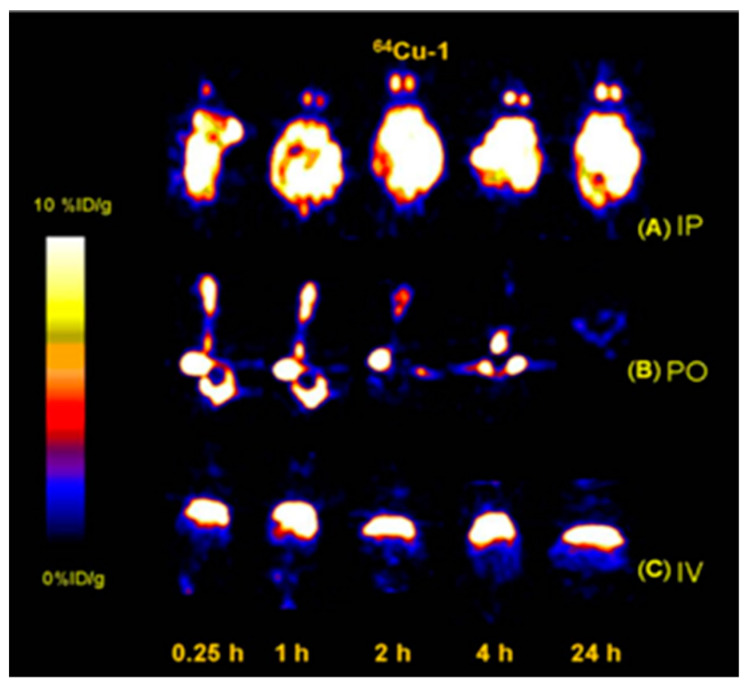
Coronal PET images of the antimicrobial peptoid ^64^Cu-1 after (**A**) intra-peritoneal (IP), (**B**) oral (PO), and (**C**) intravenous (IV) administration in Balb/C mice at different time points (n = 4). Reprinted with permission from Seo et al. (2012) [129].

To investigate antimicrobial compounds in small animals, researchers from Stanford University labeled peptoids with ^64^Cu (Table 3) and determined the biodistribution of the peptoids by PET imaging [129]. Peptoids, or oligo-N-substituted glycines, are a novel class of polymers that mimic peptides and proteins but have a more stable molecular structure and higher bioavailability compared to natural peptides [130,131]. They also display increased cell permeability and can avoid immune recognition [132,133]. After developing three novel cationic amphipathic peptoids and studying their biodistribution after intravenous injection, the first peptoid termed ^64^Cu-1 was chosen for further study after oral and intraperitoneal administration in mice. It consisted of a cationic amphipathic peptoid conjugated with N-terminal 1,4,7,10-tetraazacyclododecane-1,4,7,10-tetraacetic acid (DOTA) and labeled with ^64^Cu. Peroral ^64^Cu-1 showed only gastrointestinal activity and eventual elimination after 24 h, with almost no systemic absorption (Figure 12) [129]. 

## 4. Conclusions and Future Developments

In this review, we established that studying the oral delivery of peptides in animals is difficult and should be conducted more often with nuclear imaging techniques which are highly sensitive at low therapeutic doses, quantifies organ activity (percentage of dose per organ) in real-time, provides early physiological information, allows for easy study of disease progression, and detects abnormalities during GI transit and/or circulation reliably [137]. Commonly used techniques such as biodistribution studies, microscopy, and MRI either do not allow for real-time analysis over extended time periods or do not provide accurate quantification of peptide uptake [138]. 

**Figure 13:**
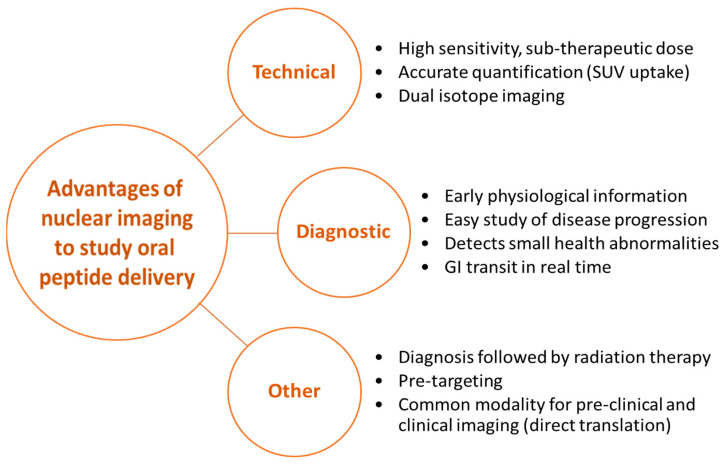
Advantages of nuclear imaging to study oral delivery of peptides.

Additional benefits of nuclear imaging include the possibility of dual isotope imaging, theranostic applications, and obtaining immediate feedback on the success of the treatment (Figure 13). Dual isotope imaging helps to concurrently image the carrier as well as the peptide after radiolabeling them with radioisotopes of different gamma radiation energies. Subsequently, the peptide uptake mechanism and DDS clearance can be investigated in vivo fully quantitatively, leading to an improved understanding of peptide pharmacokinetics [55]. In theranostic applications, α- and β-emitting radioisotopes can be orally delivered to impart site-specific radiotherapy, in combination with γ-radioisotopes that provide diagnostic information about the molecular environment and report the success of the therapeutic intervention [139]. A milestone in nuclear imaging has been the development of fusion imaging, whereby SPECT and/or PET is superimposed with CT or MRI images to display anatomical and functional pharmacological properties of peptides concurrently in the same animal over time [139]. PET and SPECT imaging thus helps to translate work from pre-clinical to clinical research and speeds up getting to first human trials. 

While nuclear imaging has many benefits, there are some limitations as well [140]. For instance, the spatial resolution of PET and SPECT imaging is lower than MRI and optical techniques; however, recent scanners are starting to bridge this gap [141]. Another drawback of nuclear imaging is the handling and exposure to ionizing radiation that requires radiation safety precautions [141]. Recent PET technology provides whole-body imaging at 40× lower radiation doses with shorter acquisition times, hence reducing potential tissue damage in humans significantly [142,143]. Furthermore, peptide pre-targeting followed by in vivo ‘click chemistry’ also reduces the radiation exposure as the peptide DDS is first delivered to the target site followed by the administration of a short half-life radioisotope which clicks covalently to the peptide in vivo, and can then be imaged or deliver treatment with minimal exposure [144]. 

In summary, oral delivery of peptides can be well studied and quantified with nuclear imaging and should be used more during early pilot studies and the development of novel peptide DDS. However, the literature review discussed in this review makes it evident that very limited nuclear imaging research has been performed until now. One major reason for this could be that the radiolabeling of drugs and DDS and its imaging in SPECT or PET cameras are rather specialized. While availability is limited, collaborating with experts in the field might yield excellent results [59]. Another reason for not using nuclear imaging to study oral peptide delivery could be that contrast of the gastrointestinal tract is low and prevents precise localization of the peptide in the different GI tract areas. The application of Artificial Intelligence (AI) is an ingenious way to solve this problem as the entire GI tract can be mapped, giving the exact location of peptide uptake, its quantification, and analysis within minutes. This is very similar to already existing techniques such as brain mapping of PET images [145]. Many recent examples from medical imaging support the notion that we are only at the beginning of gaining additional information from the use of AI, information that adds valuable data points to the trained eye of radiologists [146] and should be applied in the near future for gastrointestinal imaging. 

When faced with a plethora of parameters and challenges during oral delivery, nuclear imaging can help to understand early on the in vivo path of a novel oral peptide DDS over time in groups of small numbers of animals. Nuclear imaging thus contributes an effective and fully quantitative tool to study the pharmacokinetic and pharmacodynamic profile of oral peptide formulations.

## Figures and Tables

**Table 1:** Commercially available FDA-approved oral peptide formulations [24,25] (* discontinued after 2017, ^#^ also approved by EMA). ^^^ Sodium N-[8-(2-hydroxybenzoyl) amino caprylate.

Therapeutic Peptide	Brand	Application	Additional Components	Bioavailability
Aliskiren *	Tekturna^®^, Tekamlo^®^, Amturnide^®^	Treat hypertension		
Boceprevir ^#^ [24]	Victrelis^®^	Treat chronic hepatitis C	Protease inhibitor	Up to 65%
Colistin [26]	Koolistin^®^	Treat multi-drug resistant bacterial infections		Local
Cyclosporine [27]	Neoral^®^/Sandimmune^®^	Suppress immune system	Non-ionic surfactant	25–30%
Desmopressin [28]	DDAVP^®^ tablets, DDAVP^®^ Melt, Minrin^®^	Treat central diabetes insipidus, primary nocturnal enuresis, blood disorders	Desmopressin acetate hydrate or Arginine Vasopressin	0.08–0.16%
Glutathione	Reduced L-Glutathione	Reduce gut inflammation in cystic fibrosis patients [29]	Curcumin, no excipients [30]	Not documented
Linaclotide ^#^	Linzess^®^, Constella^®^	Treat irritable bowel syndrome [31]	Microcrystalline cellulose spheres, enteric coating, hard gelatin capsules	Local
Octreotide [32]	Mycapssa^®^	Treat acromegaly	Polyvinylpyrrolidone (PVP-12), polysorbate 80, co-emulsifiers (glyceryl caprylates), gelatin capsules, and Acryl-EZE^®^ (methacrylate)	53–88%
Ombitasvir [33]	Viekirax^®^	Treat Chronic hepatitis C	Combination of dasabuvir, ombitasvir, paritaprevir, and ritonavir	48–53%
Pancrelipase [24]	Pancrecarb^®^, Viokace^®^, ULTRESA^®^, PERTZYE^®^, ULTRASE^®^, ZENPEP^®^	Improve food digestion	Pancreatic amylase, pancreatic lipase, and chymotrypsin	27–29%
Ragweed [24]	Ragwitek^®^	Treat ragweed allergy	Pollen extract	Not documented
Sacrosidase	Sucraid^®^	Hydrolyze sucrose	Sucrase [34]	Local
Semaglutide [35,36]	Rybelsus^®^	Induce insulin secretion	Enzyme inhibitor, enteric coating, Absorption enhancer (SNAC) ^	~1%
Taltirelin [37]	Ceredist^®^ Ceredist OD^®^	Protect thyrotropin releasing hormone from enzyme hydrolysis		75%
Tyrothricin [38]	Several brands	Treat infected skin and oro-pharyngeal mucus membranes		Local
Vancomycin [39]	Vancocin^®^	Treat pseudomembranous colitis		Local

**Table 2:** Summary of SPECT radionuclides and reaction parameters used in the preparation of radiolabeled peptides for pre-clinical and clinical imaging [105,114,115,116,117]. ^a^ Hydrazinonicotinic acid; ^b^ diethylenetriaminepentaacetic acid; ^c^ 1,4,7-triazacyclononane-1,4,7-triacetic acid; ^d^ 1,4,7,10-tetraazacyclododecane-1,4,7,10-tetraacetic acid; ^e^ glucagon-like-phosphate-1 peptide. RT = room temperature.

Radionuclide	Half-Life (h)	Chelator/Prosthetic Group	Reaction Temperature	Reaction pH	Associated Preclinical/Clinical Peptide
^99m^Tc	6.01	Amidethiols, tetraamines (N4) or HYNIC ^a^	RT for N4 80–100 °C for HYNIC		Octreotide, demobesin, sulfated cholecystokinin, demogastrin, exendin
^111^In	67.31	DTPA ^b^, NOTA ^c^	RT for DTPA, 60–65 °C for NOTA [118]	4–5	Octreotide, bombesin, neuropeptide Y, AMBA, minigastrin, GLP-1 ^e^
^67^Ga	78.28	DTPA ^b^, NOTA ^c^, DOTA ^d^	RT for DTPA, NOTA 85–100 °C for DOTA	2–11	PESIN
^123^I	13.22	Bolton-Hunter reagent	RT	7–8	GLP-1

**Table 3:** Summary of PET radionuclides and reaction parameters used in the preparation of radiolabeled peptides for pre-clinical and clinical imaging [105,114,116,117,134,135,136]. ^a^ N-succinimidyl 4-[^18^F]fluorobenzoate; ^b^ 4-[^18^F]fluorobenzaldehyde; ^c^ 2-[^18^F]fluoro-2-deoxyglucose; ^d^ diethylenetriaminepentaacetic acid; ^e^ 1,4,7-triazacyclononane-1,4,7-triacetic acid; ^f^ 1,4,7,10-tetraazacyclododecane-1,4,7,10-tetraacetic acid; ^g^ 1,4,8,11-tetraazacyclotetradecane, 1,4,8,11-tetraacetic acid; ^h^ cross-bridged-cyclam with TETA; ^i^ 3,6,10,13,16,19-hexaazabicyclo [6.6.6]icosane; ^j^ N-succinimidyl-5-[*I]iodo-3-pyridine carboxylate; ^k^ N-succinimidyl-3-[*I]iodobenzoate; ^l^ Arginine-Glycine-Aspartate probe.

Radionuclide	Half-Life (h)	Chelator/Prosthetic Group	Reaction Temperature	Reaction pH	Associated Preclinical/Clinical Peptide
^18^F	1.83	FSB ^a^, FBA ^b^, FDG ^c^	RT	~7	Octreotate, bombesin analog BAY, GLP-1, RGD ^l^
^68^Ga	1.13	DTPA ^d^, NOTA ^e^, DOTA ^f^	85–100 °C for DOTA	2–4, 7–11	Somatostatin, exendin, RGD ^l^
^64^Cu	12.70	NOTA ^e^, TETA ^g^, CB-TE2A ^h^, Sar ^i^	RT for NOTA, TE2A 25–37 °C for Sar	5–8	Octreotide, bombesin, exendin, RGD ^l^
^124^I	100.22	Bolton-Hunter reagent, SIPC ^j^ or SIB ^k^	RT	7–8	GLP-1
^86/90^Y	14.74	DOTA ^f^	80–85 °C	4–5	Somatostatin, RGD ^l^
